# Preventive potential of nano silver fluoride versus sodium fluoride varnish on enamel caries like lesions in primary teeth: in vitro study

**DOI:** 10.1186/s12903-022-02271-6

**Published:** 2022-06-20

**Authors:** Dina I. El-Desouky, Azza Hanno, Yasmine Elhamouly, Sara A. Hamza, Lubna M. El-Desouky, Karin M. L. Dowidar

**Affiliations:** 1 Department of Pediatric Dentistry and Dental Public Health, Faculty of Dentistry, Alexandria University, Alexandria, Egypt; 2 Department of Pediatric and Community Dentistry, Faculty of Dentistry, Pharos University in Alexandria, Alexandria, Egypt; 3 Department of Oral Biology, Faculty of Dentistry, Alexandria University, Alexandria, Egypt; 4 Department of Pharmaceutics, Faculty of Pharmacy, Alexandria University, Alexandria, Egypt

**Keywords:** Artificial caries, Enamel demineralization, Fluoride varnish, Nano silver fluoride, Primary teeth

## Abstract

**Background:**

Professionally applied topical fluoride preparations have been commonly used and have proven to prevent dental decay. Alternative preparations that provide further benefits may be of interest to investigate. This study aimed to investigate the effect of experimental nano silver fluoride (NSF) formulation compared to commercial sodium fluoride varnish (FV) on prevention of in vitro demineralization of initially sound enamel in primary teeth.

**Methods:**

Forty-eight extracted/exfoliated sound molars were sectioned buccolingually into 96 specimens then assigned randomly into two equal groups. Each group was further subdivided into two equal subgroups (Ia: NSF, IIa: FV, Ib and IIb as negative controls). The test materials were applied, then all the specimens were subjected to a demineralization pH cycling model for 7 days. Specimens were examined for surface microhardness using Vickers microhardness device and lesion depth was evaluated by polarized light microscope using image J 1.46r software. Data were analyzed using paired t-test, independent t-test, and Mann Whitney U test.

**Results:**

The test materials were significantly superior to their negative controls, (*P* < 0.001) and comparable to each other, (*P* > 0.05) regarding microhardness and lesion depth. In comparison to FV, NSF showed lower yet statistically insignificant percent increase in microhardness and decrease in lesion depth, (*P* = 0.81, 0.86, respectively). Qualitative evaluation revealed that both agents reduced the lesion depth formation.

**Conclusion:**

NSF showed similar effect to that of FV in limiting in vitro enamel demineralization caused by acidic challenge. Hence, it could be regarded as a promising alternative preventive agent in primary teeth.

**Supplementary Information:**

The online version contains supplementary material available at 10.1186/s12903-022-02271-6.

## Background

Dental caries is still a major health problem despite dental care improvements [1]. Repair of carious primary teeth is excessively time‑consuming, costly, and challenging [2].Considering the enormity of this problem and its effect on the children’s quality of life, prevention should be a prime concern of the dental profession [2]. Primary prevention of caries or demineralization resistance is achieved via using various fluorides [3]. Professionally applied topical fluoride varnish (FV) is a prominent successful agent amongst the available anti-caries agents [2, 4]. Its application two to four times a year would substantially reduce tooth decay in moderate and high-risk children [5]. However, it is technique sensitive and costly on the individual and community levels as it requires multiple applications per year [6, 7]. Moreover, concerns about ingestion, toxicity and dental fluorosis have been aroused by scientific research [8]. Owing to these drawbacks, seeking alternatives could be valuable.

Nano silver fluoride is another promising anti-caries agent. It is an experimental formula that has combined the preventive and antimicrobial properties of nano silver particles and fluoride [9]. It is obtainable as a reddish yellow solution containing silver nanoparticles (AgNPs), chitosan and fluoride [10]. Various studies have shown that chitosan and AgNPs have excellent antimicrobial properties against mutans streptococci and lactobacilli, which are the primary cariogenic promoting pathogens [9, 10]. Fluoride significantly reduces the bacterial extracellular polysaccharide formation and interferes with bacterial enzyme activity [11]. The caries arrest effectiveness of NSF was explained by the synergism of the components of its formulation [12]. It is claimed to be safe [11], stable for three years, cost-effective, ecofriendly, easy to use and could be applied annually [9, 12]. It is also worth mentioning that this simple, non-invasive, less technique sensitive material provides a great scope for its use in public health programs [13].

Although data on NSF have shown promising results in arresting [12, 14] and remineralizing pre-formed or pre-existing carious lesions [10, 15], to the best of our knowledge, no published studies have investigated its effect on resisting demineralization of initially sound enamel in primary teeth. Therefore, the present in vitro study aimed to investigate the potential of experimental NSF formulation in prevention of demineralization of sound enamel compared to commercial 5% sodium fluoride varnish in primary teeth. The proposed hypothesis was that NSF and sodium fluoride varnish would have similar preventive effect on the enamel surface microhardness (SMH) and lesion depth formation after artificial acidic challenge in sound primary teeth.

## Materials and methods

This comparative in vitro investigation was approved by the Research Ethics Committee at the Faculty of Dentistry, Alexandria University, Egypt (IRB 00010556–IORG 0008839), prior to commencement. The minimal sample size was calculated based on a previous study by Nozari [15] using G*power 3.0.10. A total sample of 40 specimens was required based on 5% alpha error. 80% power, and standardized effect size (δ) of 0.915 [16, 17].

Forty-eight human primary molars, whether normally exfoliated or extracted for serial extraction purposes [18], were collected from the outpatient clinic of the Pediatric Dentistry Department, Faculty of Dentistry, Alexandria University, Egypt. They were cleaned carefully from blood and debris then examined using a magnifying lens [19] to ensure that they met the inclusion criteria. Teeth were included if they had no caries, previous fillings, cracks, or developmental anomalies. They were then stored in 2% formaldehyde at room temperature till required for use [20].

Randomization sequence in blocks of 4 was created by a trial independent person using random allocation software version 1.0.0 [21], to allocate the teeth to one of the 2 main groups. A second randomization list was generated for each group to allocate each half into one of the 2 subgroups. The allocated subgroup was written on a piece of paper that was folded and kept in opaque sealed envelopes. At the time of intervention, the subgroup to which the tooth was allocated to, was identified by the trial independent personnel [15].

### Sample preparation

The selected teeth were cleaned with fluoride free pumice then washed with distilled water and air-dried. A 4 × 4 mm square of self-adhesive tape was stuck at the center of the middle third of the buccal surface of each tooth [22]. All surfaces of the teeth were coated with a layer of acid resistant nail varnish. After drying, the self-adhesive tapes were removed leaving only a window of 4 × 4 mm of enamel exposed in each tooth [23]. Each tooth was mounted in a self-cure acrylic resin inside a cylindrical plastic mold with its buccal surface facing upwards [24]. Each tooth was sectioned buccolingually into two equal halves [25]. All the specimens were re-coated with nail varnish to cover any surface that has been exposed due to sectioning as well as the cut surface [15].

### Nano silver fluoride preparation

Preparation of NSF was carried out according to the method described by Targino et al. [9]. Synthesis of aqueous solution of silver nanoparticles was carried out via chemical reduction of silver nitrate with sodium borohydride and chitosan biopolymer as a carrier for the silver nano particles to improve the molecular weight and stabilize the compound. Chitosan (28.7 ml, 2.5 mg/ml) was first dissolved in 1% acetic acid by stirring overnight on a magnetic stirrer. Then, the chitosan mixture was filtered through a vacuum filter unit into a flask and transferred to an ice-cold bath. Under vigorous stirring, silver nitrate (1 ml, 0.11 mol/L) was added to the above mixture, then freshly prepared sodium borohydride (0.3 ml, 0.8 mol/L) was added drop by drop. The reduction of Ag+ was initiated immediately as the solution changed from colorless to light yellow and ended up reddish. The flask was then removed from the ice bath and the sodium fluoride (10,147 ppm of fluorine) was incorporated [9] to enhance the antibacterial effect of the compound in addition to favoring remineralization and inhibiting demineralization which occur continuously in the oral environment [26].

### Characterization of NSF

**Fig. 1:**
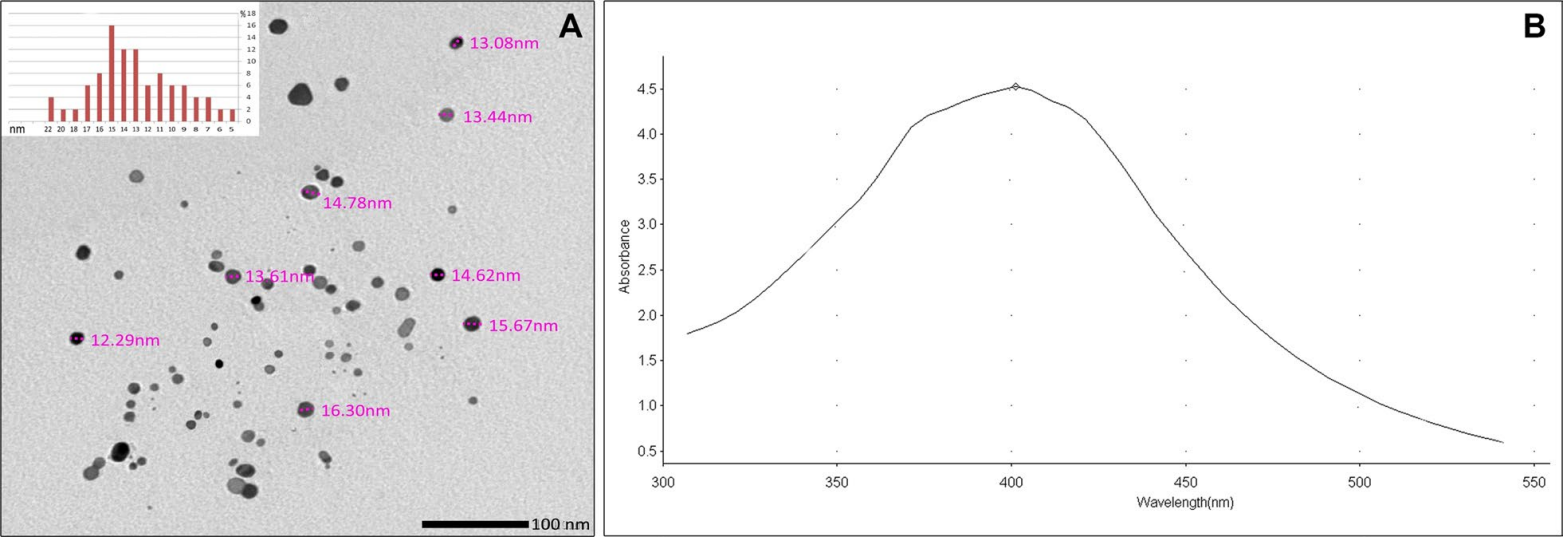
**A** Transmission electron microscopy of the prepared NSF specimen showing the shape and size of silver nanoparticles and a histogram showing percentage distribution of silver nanoparticles in the same specimen. **B** Ultraviolet- visual spectrum of synthesized silver nanoparticles

The size and morphology of AgNPs was characterized using field emission transmission electron microscope (TEM) (JEOL JEM-2100F) [10]. The electron micrographs showed that most of the particles exhibited a spherical shape and more than 50% of the detected particles' size ranged from 13 to 16 nm (Fig. 1a). Silver nano particles were also validated using UV/Vis spectrophotometer (Thermo Electron- Evolution 300) to detect the UV/Vis absorption spectrum of the resulting solution [14, 27] (Fig. 1b). The tested specimen exhibited a peak at 401 nm wavelength, denoting the presence of AgNPs with average size of 15 nm [28].

### Grouping and application of the preventive agents (Fig. 2)

**Fig. 2:**
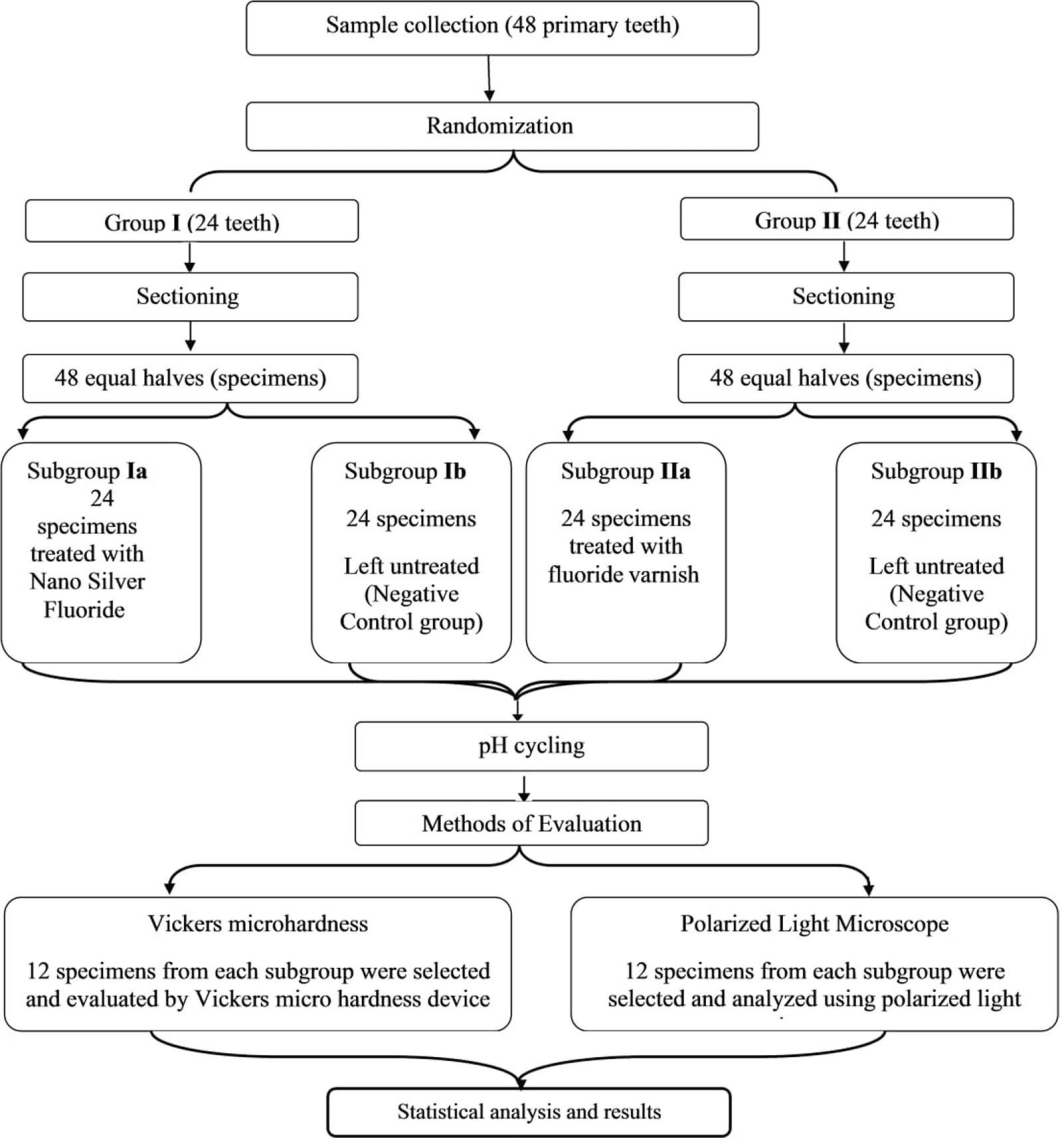
Flow chart of the study design

Group I (NSF): in subgroup Ia, twenty-four specimens were treated with NSF. Each specimen received 2 drops of NSF solution using a micro brush on the exposed enamel windows of the buccal surfaces. The solution was left in contact with tooth surface for 2 min then rinsed with a flow of distilled water [15]. In subgroup Ib, the twenty-four specimens were left untreated to serve as negative controls. Group II (FV): in subgroup IIa, twenty-four specimens were treated with 5% sodium fluoride varnish (Profluorid ®, VOCO, Germany) according to the manufacturer's instructions. A thin uniform layer of fluoride varnish was applied to the exposed enamel windows using a disposable brush. All the specimens were stored in artificial saliva for 24 h [15]. Afterward, the fluoride varnish was removed from the specimens' surfaces with a cotton swab soaked in acetone [24]. In subgroup IIb, twenty-four specimens were left untreated to serve as negative controls.

### Acidic challenge

An in vitro demineralization pH cycling model was used in this study. Over a period of seven days, all the specimens were subjected to 5 pH cycles at 37 °C followed by 2 days of remineralization to preserve the enamel surface layer allowing accurate surface microhardness determination [29, 30]. Each specimen was stored in a separate container. Specimens were immersed in the demineralizing solution (calcium and phosphate, both 2.0 mmol/L, in 75 mmol/L acetate buffer, pH 4.7; 0.04 µg F/mL, 2.2 mL/mm2) for 3 h, followed by distilled water rinse. Then, they were immersed in the remineralizing solution (1.5 mM CaCl2, 0.9 mM NaH2PO4, 0.15 M KCL had a pH of 7.0, 1.1 ml/mm2) for 21 h [31, 32].

### Outcome assessment

The preventive effect of NSF and FV was assessed by evaluating the specimens quantitatively and qualitatively. Quantitative assessment of the enamel surface microhardness was performed using Vickers microhardness device (Wilson microhardness tester, Japan) and assessment of the enamel lesion depth was performed by polarized light microscope (PLM) (Olympus America Inc.). Qualitatively, the extent of the lesion from the enamel surface into the depth of enamel, the presence or absence of Hunter Shreger Bands and the surface prismless layer of enamel were assessed using the PLM. The outcome assessors were blinded to the treatment type.

#### Microhardness test evaluation

After pH cycling, twelve specimens from each test subgroups (Ia, IIa) and their corresponding controls (Ib, IIb) (n = 48) were examined with Vickers microhardness device with a load of 50 gm for 10 s onto the surface of each specimen to make impression on the specimen surface. After load removal, the diagonals of the resulting impression were measured by built in scaled microscope. This measurement was converted into a hardness number using the following equation: HV = 1854 P/d2, where HV is the Vickers number, *P* is the applied load and d is the length of the diagonals. Three indentations were made in the enamel surface of each specimen then the mean was calculated and considered as the hardness number of the specimen [33].

#### Polarized light microscope evaluation

##### Specimens’ preparation

**Fig. 3:**
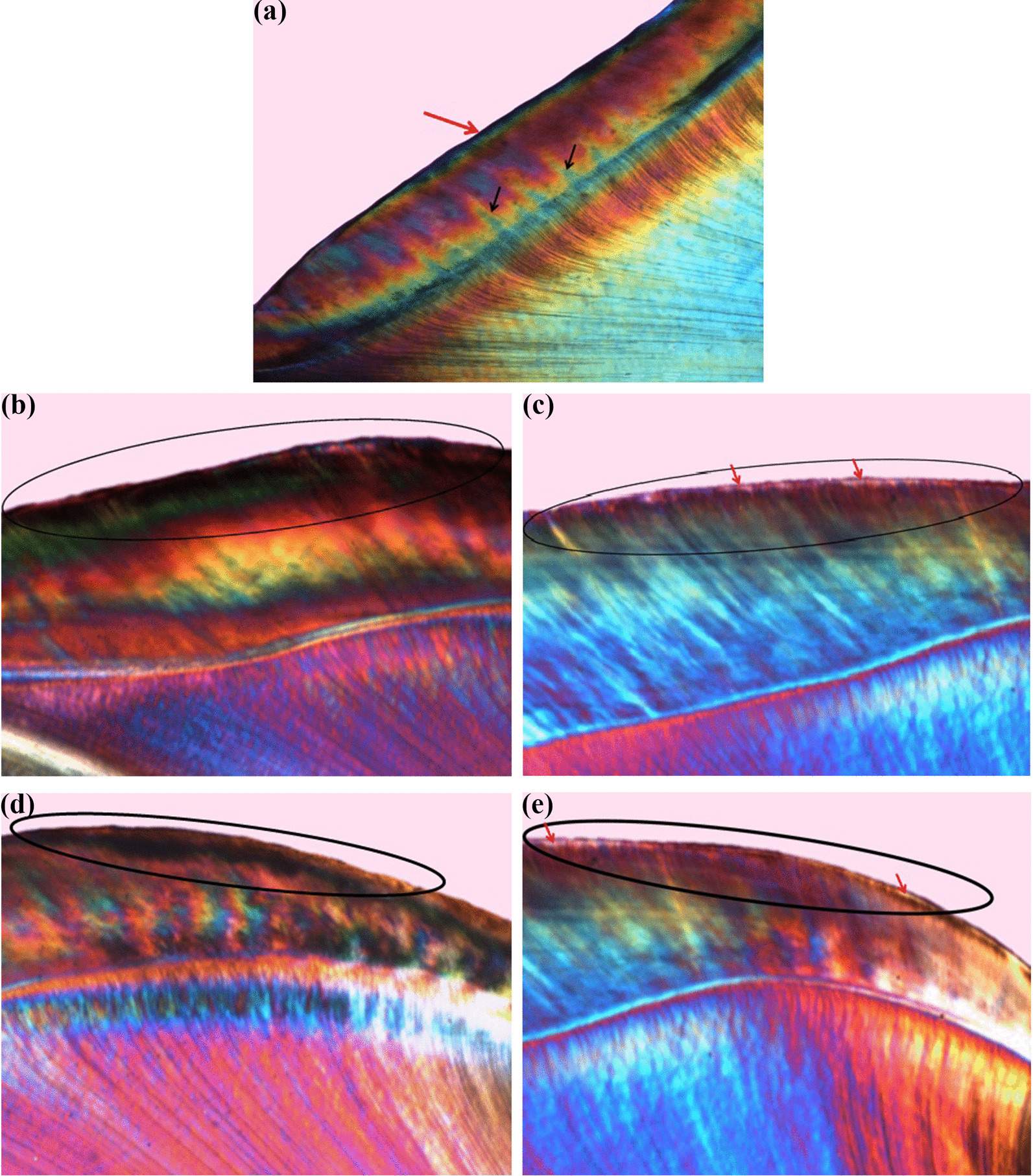
Polarized light photomicrograph of a longitudinal ground section of: **a.** Normal enamel with HSBs (black arrows) and prismless surface layer (red arrows). **b.** Control specimen (Ib) showing evident dark demineralized enamel band with high degree of positive birefringence (circle). **c.** Enamel treated with NSF (Ia) showing prominent lesion depth reduction with noticeable negative birefringence (circle) and surface mineralization (red arrows). **d**. Control specimen (Ib) showing demineralized enamel band (circle). **e**. Enamel treated with NSF (Ia) showing remarkable protection against demineralization (circle) with negative birefringent surface and remineralization band (red arrows), magnification ×40

**Fig. 4:**
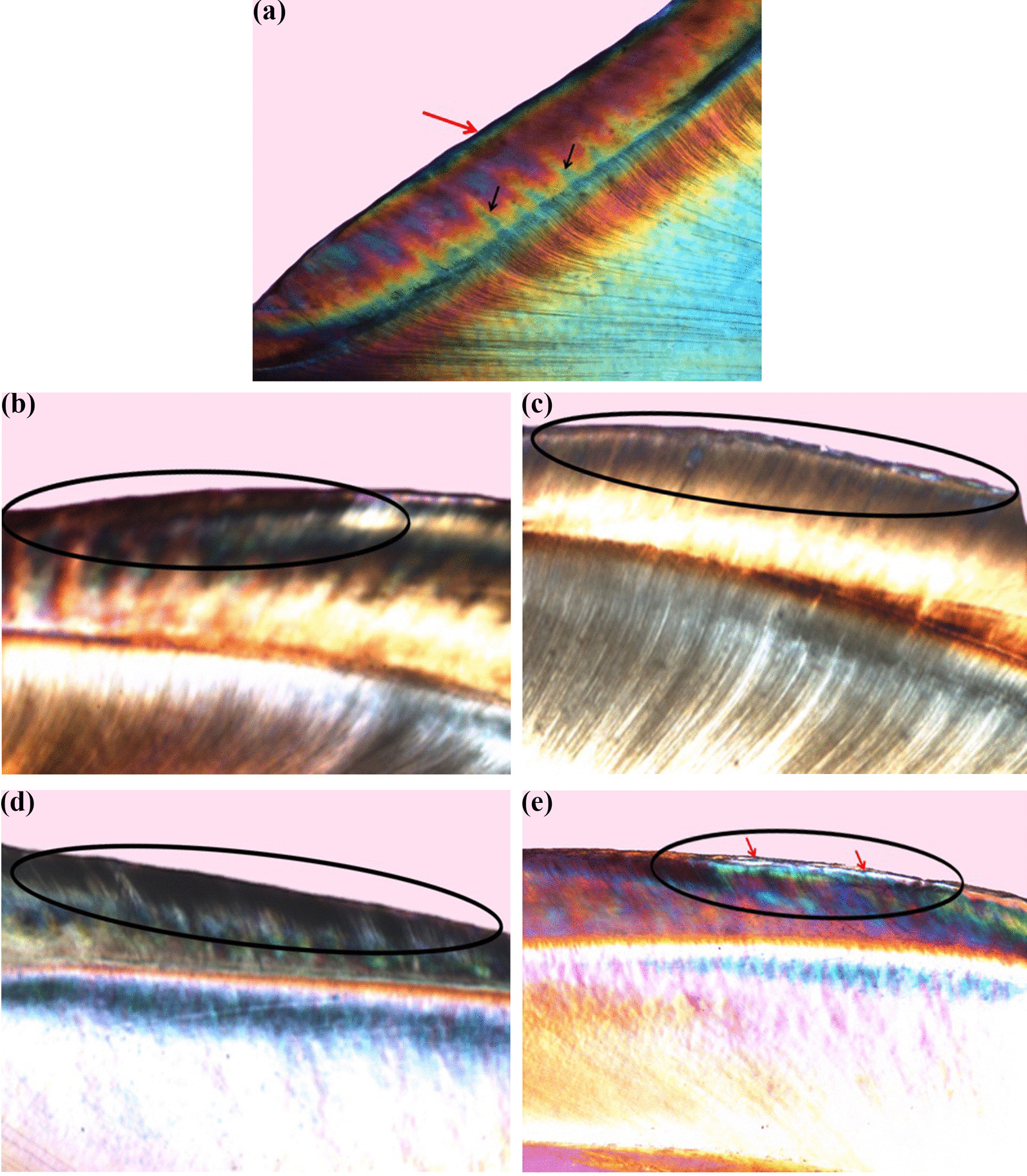
Polarized light photomicrograph of a longitudinal ground section of: **a.** Normal enamel. **b**. Control specimen (IIb) showing a deep dark demineralization band. **c.** Enamel treated with FV (IIa) showing slight decrease in the lesion extent. **d**. Control specimen (IIb) showing high degree of positive birefringence with loss of typical structural enamel features within the lesion body. **e.** Enamel treated with FV (IIa) showing obvious lesion limitation with evident mineralized surface layer (red arrows), magnification ×40

The remaining twelve specimens from each test subgroup (Ia, IIa) and their corresponding controls (Ib, IIb) (n = 48) were prepared for analysis by PLM. Longitudinal ground sections of about 15 μm thickness were prepared and then mounted using Canada balsam to hold the specimen in place between the slipcover and the glass slide. A ground longitudinal section of normal enamel (Figs. 3a, 4a) of an untreated tooth was prepared as previously described to be compared with the sections included in the study as a reference section [34].

##### Quantitative lesion depth measurement

The sub-surface depth measurements were done using image J1.46r software (National Institutes of Health, USA). The readings by the software were represented in pixel unit. To turn the pixel unit into millimeter (mm) unit, a photomicrograph was taken to a known 2 mm graduated slide of Olympus microscope with the same magnification of all specimens (40×) as a reference slide. The reference slide was projected onto the computer image processing software (Image J1.46r) as a scale. After setting the scale by the software to get the readings in mm, the results were all recorded in micrometers (μm) by multiplying the numbers in thousand. The mean depth of the enamel lesion of each specimen was measured by averaging three lines: one at each side and one at the center of the lesion within the subsurface of the lesion body, perpendicular to the outer layer of the enamel surface and extending to the translucent band [34].

##### Qualitative histological evaluation

All prepared sections were examined by PLM to evaluate the changes in enamel after pH cycling. Photomicrographs were taken with a digital camera with magnification of 40× (eyepiece 10× & objective lens 4×) to achieve comparison in histological features between the study and control specimens. They were also compared to the reference section.

### Statistical analysis

Data were analyzed using IBM SPSS for Windows version 23.0 (IBM Corp., Armonk, N.Y., USA). Normality was checked using descriptive statistics, plots (histogram and box plot) and Shapiro Wilk test. Normally distributed data was presented using Mean ± SD, while median and inter quartile range (IQR) were used for non-normally distributed data. Differences in lesion depth and surface microhardness between test and control in NSF and FV groups were analyzed using paired t-test, while differences between test subgroups were analyzed using independent t-test. Percent change in surface microhardness and lesion depth were assessed using Mann Whitney U test and independent t test, respectively. Percent change was calculated according to the formula [(Values after treatment (test)—values after demineralization (control)/values after demineralization (control)] × 100 [35].

## Results

Surface microhardness (Mean ± SD) of all specimens is shown in Table 1. The microhardness in the test subgroups Ia: NSF (312.75 ± 26.35 VHN) and Ib: FV (309.27 ± 22.53 VHN) was significantly higher than their negative controls IIa and IIb (301.50 ± 26.95 VHN and 293.69 ± 23.24 VHN), respectively (*P* < 0.001). In comparison to Ib: FV, the Ia: NSF subgroup had insignificantly higher absolute microhardness values (*P* = 0.73). Additionally, NSF showed lower yet insignificant percent increase in microhardness by (median = 3.23%) than the FV (median = 4.17%), (*P* = 0.81).

**Table 1:** Vickers microhardness values (VHN) of all studied groups and percent difference between each test and its control subgroup

	NSF (subgroup Ia) (n = 24)		FV (subgroup IIa) (n = 24)	
	Ia (test) (n = 12)	Ib (control) (n = 12)	IIa (test) (n = 12)	IIb (control) (n = 12)
Mean ± SD	312.75 ± 26.35	301.50 ± 26.95	309.27 ± 22.53	293.69 ± 23.24
Paired t test (*P* value)	6.40 (< 0.001*)		5.35 (< 0.001*)	
Independent t test (*P* value)	0.347 (0.73)			
Percent increase				
Median	3.23		4.17	
IQR	2.54–4.39		3.18–6.37	
MWU (*P* value)	− 1.328 (0.81)			

*NSF* Nano silver fluoride, *FV* Fluoride varnish, *IQR* Interquartile range, *MWU* Mann Whitney U test

*Statistically significant at *P* value ≤ 0.05

Lesion depth values are shown in Table 2. The lesion depth of subgroup Ia: NSF was significantly lower than that of subgroup Ib: control with mean values of 244.03 ± 79.73 µm, and 384.30 ± 110.91 µm, respectively (*P* < 0.001). Similarly, a significant difference was observed in the mean lesion depth between subgroup IIa: FV (262.73 ± 99.65 µm) and IIb: control (416.96 ± 107.42 µm) (*P* < 0.001). The test subgroup Ia: NSF had a lesser mean lesion depth than IIa: FV with no statistically significant difference (*P* = 0.61). The NSF group had a 36.36 ± 9.54% decrease in lesion depth, whereas, the FV group had a 37.30 ± 16.67% reduction. The two materials, however, showed no statistically significant difference (*P* = 0.86).

**Table 2:** Lesion depth values (µm) of all studied groups and percent difference between each test and its control subgroup

	NSF (subgroup Ia) (n = 24)		FV (subgroup IIa) (n = 24)	
	Ia (test) n = 12)	Ib (control) (n = 12)	IIa (test) n = 12	IIb (control) (n = 12)
Mean ± SD	244.03 ± 79.73	384.30 ± 110.91	262.73 ± 99.65	416.96 ± 107.42
Paired t (*P* value)	− 8.56 (< 0.001*)		− 6.65 (< 0.001*)	
Independent t test (*P* value)	− 0.507 (0.61)			
Percent Reduction				
Mean ± SD	36.36 ± 9.54		37.30 ± 16.67	
Independent t test (*P* value)	0.169 (0.86)			

*NSF* Nano silver fluoride, *FV* Fluoride varnish

*Statistically significant at *P* value ≤ 0.05

Regarding polarized light microscope evaluation, the normal enamel specimen showed normal course of enamel rods with alternative Hunter–Schreger Bands (HSBs) reflecting normal mineralization and birefringence of enamel. It also showed prismless surface layer appearing as a continuous ribbon all over the enamel surface with no structural details (Figs. 3a, 4a).

The protective effect of NSF was observed by the outstanding reduction in the lesions’ depth seen in subgroup Ia (Fig. 3c, e) compared to subgroup Ib (Fig. 3b, d). The negative birefringence emphasized the effect of this treatment. Most of the specimens showed a surface layer that appeared more mineralized than the deeper areas. Almost all the specimens in subgroup Ib (Fig. 3b, d) showed evident dark bands starting from the enamel surface and proceeded inward circumscribing the created lesions and reflecting a high degree of positive birefringence with loss of HSBs within the lesion body (magnification 40×). The protective effect of FV was observed by the noticeable reduction in the lesions' depth in subgroup IIa (Fig. 4c) compared to IIb. A highly mineralized surface layer was noted in most of the specimens (Fig. 4e). The untreated specimens in subgroup IIb showed similar observations as in subgroup Ib. A relatively high degree of positive birefringence with loss of the typical structural features of enamel was seen within the body of the lesion (Fig. 4b, e).

## Discussion

In this study, the potential of experimental NSF formulation to resist enamel demineralization caused by acidic challenge was compared to that of commercial 5% sodium fluoride varnish in primary teeth. The obtained results revealed that both agents have similar effect in controlling the carious lesion formation. Thus, the null hypothesis was accepted. In line with these results, Teixeira et al. [10] showed that NSF was more effective in decreasing enamel demineralization after pH cycling than the negative control sample.

Fluoride varnish was regarded as a commercial positive control because it is one of the most studied preparations with proved safety and efficacy in preventing dental caries [36]. Application of FV in the current study confirmed its ability to decrease demineralization in the treated specimens in which the mean microhardness value of the treated specimens was about 5% higher than that of the untreated specimens, and the lesion depth in FV treated specimens was 37% lower than that of the untreated specimens [24].

The fluorine content in the NSF used in the current study was 10,147 ppm, whereas that of the FV was 22,600 ppm. In addition, the contact time of NSF with tooth structure was only two minutes, while it was 24 h with the FV. Regardless of the variation that might seem in favor of the FV, NSF showed similar preventive capacity to FV with no statistically significant difference.

The protective effect of NSF could be explained by the very small particle size of AgNPs which facilitated the penetration of the material into the enamel structure maximizing its effect [37]. Correspondingly, Teixeira et al. [10] reported no statistically significant difference between NSF and sodium fluoride (NaF) dentifrices in preventing enamel demineralization although NaF has shown lower percentage of microhardness variation. This could be explained by the different pH cycling protocol used and the repeated application of dentifrice slurries before each pH cycle.

On the contrary, the study by Nozari [15] revealed that the specimens treated with NSF had the highest surface microhardness values compared to fluoride varnish and nano hydroxyapatite paste. This result could be related to the different study design which focused on comparing remineralization of the pre-formed enamel caries lesion not the ability of the tested materials to prevent demineralization of sound enamel as in the current study.

The results of the present study revealed that the subgroups which had the highest microhardness values (Ia and IIa) had the lowest lesion depth values and vice versa. This might be attributed to the formation of a highly mineralized surface layer that resulted from direct contact with materials having high fluoride content [38]. In addition, the pH cycling model that was used in the present study had the purpose of preserving the enamel surface layer and create a sub-surface lesion that closely imitates the natural incipient carious lesion [29, 39]. This behavior might have led to a surface layer that had a relatively high microhardness value and a sub-surface lesion that was evident in the polarized light microscope.

Qualitative evaluation was performed using polarized light microscope as it is the most sensitive and descriptive-analytical technique for evaluating the histological changes in the zones of caries like lesions [40]. In the current study, results from histological evaluation go in line with the changes in the microhardness values at the different phases. It was noted that all treated specimens in both groups showed a noticeable decrease in the extent of the lesion with a reduction in the positive birefringence of the body of the lesion in comparison to the negative control specimens. This was in agreement with Nozari [15] and Dos Santos et al. [12] who reported that the enamel treated with NSF showed shallower lesion depth and the caries was arrested compared to untreated enamel.

The current study also demonstrated that the quality of the lesions appeared different in the treated specimens as most of them showed a highly mineralized surface layer (negatively birefringent) that was not evident in the non-treated specimens. This is mostly attributed to the effect of the high fluoride content provided by both treatment materials on the specimens’ surface. In addition, the application of the treatment materials also led to the formation of calcium fluoride (CaF_2_) like layer that acted as a reservoir during demineralization periods and slowly released fluoride that protected the enamel surface against dissolution. During remineralization periods, fluoride attracted calcium and phosphate ions present in the remineralizing solution forming a highly mineralized layer on the surface of the caries like lesion [41].

This designed in vitro study addressed the primary caries prevention level in which an initially sound host (human primary teeth) was used to investigate the impact of NSF on enhancing the resistance to demineralization caused by acidic challenge. To the best of our knowledge, the studies available in the literature on primary teeth are mainly focused on secondary caries prevention in which the experimental agents were tested for their attempts to repair/ remineralize the pre-formed enamel caries like lesions.

In order to mimic the clinical conditions, we used a pH-cycling model. However, the in vitro study might not reproduce the results of an in vivo one as it might not provide insights into various aspects of the caries process. The limitations of this study were that the oral factors including biofilm, oral flora, different salivary components, individuals’ dietary habits and oral hygiene practices could not be considered. In addition, the present study was limited to a period of 7 days, while the dynamic de/remineralization processes are long-term processes.

Therefore, it would be interesting for future studies to design pH-cycling models as close as possible to in vivo conditions in which the solutions mimic plaque fluid ionic concentration and pH in individuals with different caries risk situations. Additionally, the models could include the addition of organic salivary components and brushing conditions. Moreover, to investigate the dose–response of NSF in situ as well as its association with other anticaries agents and its safety in the clinical setting.

## Conclusions

Nano silver fluoride showed similar effect to that of FV on limiting enamel demineralization caused by artificial cariogenic challenge. Hence, it could be regarded as an alternative preventive measure to FV in primary teeth.

## Supplementary Information

**Additional file 1.** Study data.

## Data Availability

All the data generated or analyzed during this study are included in this published article and its Additional file 1.

## Abbreviations

NSF: Nano silver fluoride
FV: Fluoride varnish
AgNPs: Silver nanoparticles
SMH: Surface microhardness
PLM: Polarized light microscope
HSBs: Hunter–Schreger Bands
NaF: Sodium fluoride
CaF_2_: Calcium fluoride

## References

[1] Moynihan P (2016). Sugars and dental caries: evidence for setting a recommended threshold for intake. Adv Nutr.

[2] Mishra P, Fareed N, Battur H, Khanagar S, Bhat M, Palaniswamy J (2017). Role of fluoride varnish in preventing early childhood caries: a systematic review. J Dent Res.

[3] Horst JA, Tanzer JM, Milgrom PM (2018). Fluorides and other preventive strategies for tooth decay. Dent Clin N Am.

[4] Øgaard B, Seppä L, Rolla G (1994). Professional topical fluoride applications—clinical efficacy and mechanism of action. Adv Dent Res.

[5] Marinho VC, Higgins JP, Logan S, Sheiham A (2003). Topical fluoride (toothpastes, mouthrinses, gels or varnishes) for preventing dental caries in children and adolescents. Cochrane Database Syst Rev..

[6] Schwendicke F, Splieth CH, Thomson WM, Reda S, Stolpe M, Foster PL (2018). Cost-effectiveness of caries-preventive fluoride varnish applications in clinic settings among patients of low, moderate and high risk. Community Dent Oral Epidemiol.

[7] Schwendicke F, Stolpe M (2017). In-office application of fluoride gel or Varnish: cost-effectiveness and expected value of perfect information analysis. Caries Res.

[8] Vaikuntam J (2000). Fluoride varnishes: Should we be using them?. Pediatr Dent J.

[9] Targino AGR, Flores MAP, Dos Santos VE, De Godoy Bené Bezerra F, De Lun Freire H, Galembeck A (2014). An innovative approach to treating dental decay in children. A new anti-caries agent. J Mater Sci..

[10] Teixeira JA, Costa E Silva AV, Dos Santos VE, De Melo PC, Arnaud M, Lima MG (2018). Effects of a new nano-silver fluoride-containing dentifrice on demineralization of enamel and streptococcus mutans adhesion and acidogenicity. Int J Dent..

[11] Lussi A, Hellwig E, Klimek J (2012). Fluorides—mode of action and recommendations for use. Schweiz Mon Schr Zahnmed.

[12] Dos Santos VE, Filho AV, Ribeiro Targino AG (2014). A new, “Silver-Bullet” to treat caries in children—Nano Silver Fluoride: a randomised clinical trial. J Dent.

[13] Roshni RS, Shetty PJ (2020). Nano silver fluoride for arresting dental caries. J Pharm Sci Res.

[14] Puppala N, Nagireddy VR, Reddy D, Kondamadugu S, Mareddy A, Chris A (2019). Nanosilver fluoride—a paradigm shift for arrest in dental caries in primary teeth of schoolchildren: a randomized controlled clinical trial. Int J Clin Pediatr Dent.

[15] Nozari A (2017). Impact of nano hydroxyapatite, nano silver fluoride and sodium fluoride varnish on primary enamel remineralization: an in vitro study. J Clin Diagn Res..

[16] Faul F, Erdfelder E, Lang AG, Buchner A (2007). G*Power 3: a flexible statistical power analysis program for the social, behavioral, and biomedical sciences. Beh Res Methods.

[17] Pannucci CJ, Wilkins EG (2010). Identifying and avoiding bias in research. Plast Reconstr Surg.

[18] Muhamad AH, Watted N (2019). Serial extraction in orthodontics. Int J Appl Dent Sci.

[19] Ogundare TO, Ajayi DM, Idon PI, Bamise CT, Oginni AO, Esan TA (2020). Prevalence and Distribution of Cracked Posterior Teeth among Adult Patients. Open J Stomatol.

[20] Delbem ACB, Bergamaschi M, Sassaki KT, Cunha RF (2006). Effect of fluoridated varnish and silver diamine fluoride solution on enamel demineralization: pH-cycling study. J Appl Oral Sci.

[21] Saghaei M (2004). Random allocation software for parallel group randomized trials. BMC Med Res Methodol.

[22] Santos LD, Reis JI, Medeiros MP, Ramos SM, Araújo JM (2009). In vitro evaluation of fluoride products in the development of carious lesions in deciduous teeth. Braz Oral Res..

[23] De Gauw JH, Costa LMM, Silva RN, Santos NB, Tenorio MDH (2017). Evaluation of the effect of ferrous sulfate on enamel demineralization of human deciduous teeth: an in vitro study. Rev Bahiana Odontol..

[24] Mohammadi N, Farahmand FM (2018). Effect of fluoridated varnish and silver diamine fluoride on enamel demineralization resistance in primary dentition. J Indian Soc Pedod Prev Dent.

[25] Duggal MS, Toumba KJ, Amaechi BT, Kowash MB, Higham SM (2001). Enamel demineralization in situ with various frequencies of carbohydrate consumption with and without fluoride toothpaste. J Dent Res.

[26] Featherstone JDB (2000). The science and practice of caries prevention. J Am Dent Assoc..

[27] Wei D, Sun W, Qian W, Ye Y, Ma X. The synthesis of chitosan-based silver nanoparticles and their antibacterial activity. Carbohydr Res. 2009;344(17):2375–82. 10.1016/j.carres.2009.09.001. 10.1016/j.carres.2009.09.001 19800053

[28] Agnihotri S, Mukherji S, Mukherji S (2014). Size-controlled silver nanoparticles synthesized over the range 5–100 nm using the same protocol and their antibacterial efficacy. RSC Adv.

[29] De Mello Vieira AE, Botazzo Delbem AC, Takebayashi Sassaki K, Rodrigues E, Cury JA, Cunha RF (2005). Fluoride dose response in pH-cycling models using bovine enamel. Caries Res.

[30] Akyildiz M, Sönmez IS (2019). Comparison of remineralising potential of nano silver fluoride, silver diamine fluoride and sodium fluoride varnish on artificial caries: an in vitro study. Oral Health Prev Dent.

[31] Ten Cate JM, Duijsters PPE (1982). Alternating demineralization and remineralization of artificial enamel lesions. Caries Res.

[32] Buzalaf MAR, Hannas AR, Magalhães AC, Rios D, Honório HM, Delbem ACB (2010). pH-cycling models for in vitro evaluation of the efficacy of fluoridated dentifrices for caries control: strengths and limitations. J Appl Oral Sci.

[33] Cury JA, Do Amaral RC, Tenuta LMA, Del Bel Cury AA, Tabchoury CPM (2010). Low-fluoride toothpaste and deciduous enamel demineralization under biofilm accumulation and sucrose exposure. Eur J Oral Sci.

[34] De Campos PH, Sanabe ME, Rodrigues JA (2015). Different bacterial models for in vitro induction of non-cavitated enamel caries-like lesions: microhardness and polarized light miscroscopy analyses. Microsc Res Tech.

[35] Barve D, Dave P, Gulve M (2021). Assessment of microhardness and color stability of micro-hybrid and nano-filled composite resins. Niger J Clin Pract.

[36] American Academy of Pediatric Dentistry (2013). Guideline on fluoride therapy. Pediatr Dent.

[37] Corrêa JM, Mori M, Sanches HL, da Cruz AD, Poiate E, Poiate IAVP (2015). Silver nanoparticles in dental biomaterials. Int J Biomater.

[38] Tenuta LMA, Cury JA (2010). Fluoride: its role in dentistry. Braz Oral Res.

[39] Comar LP, Souza BM, Gracindo LF, Buzalaf MAR, Magalhaes AC (2013). Impact of experimental nano-HAP pastes on bovine enamel and dentin submitted to a pH cycling model. Braz Dent J.

[40] Arends J, Ten Bosch JJ (1992). Demineralization and remineralization evaluation techniques. J Dent Res.

[41] Pollick H (2018). The role of fluoride in the prevention of tooth decay. Pediatr Clin N Am.

